# Tissue-specific epigenetic inheritance after paternal heat exposure in male wild guinea pigs

**DOI:** 10.1007/s00335-020-09832-6

**Published:** 2020-04-13

**Authors:** Alexandra Weyrich, Selma Yasar, Dorina Lenz, Jörns Fickel

**Affiliations:** 1 Leibniz Institute for Zoo and Wildlife Research (IZW), Alfred-Kowalke-Str. 17, 10315 Berlin, Germany; 2 Institute for Biochemistry and Biology, University of Potsdam, Karl-Liebknecht-Str. 24- 25, 14476 Potsdam, Germany

## Abstract

External temperature change has been shown to modify epigenetic patterns, such as DNA methylation, which regulates gene expression. DNA methylation is heritable, and as such provides a mechanism to convey environmental information to subsequent generations. Studies on epigenetic response to temperature increase are still scarce in wild mammals, even more so studies that compare tissue-specific epigenetic responses. Here, we aim to address differential epigenetic responses on a gene and gene pathway level in two organs, liver and testis. We chose these organs, because the liver is the main metabolic and thermoregulation organ, and epigenetic modifications in testis are potentially transmitted to the F2 generation. We focused on the transmission of DNA methylation changes to naive male offspring after paternal exposure to an ambient temperature increase of 10 °C, and investigated differential methylated regions of sons sired before and after the paternal exposure using Reduced Representation Bisulfite Sequencing. We detected both a highly tissue-specific epigenetic response, reflected in genes involved in organ-specific metabolic pathways, and a more general regulation of single genes epigenetically modified in both organs. We conclude that genomes are context-specifically differentially epigenetically regulated in response to temperature increase. These findings emphasize the epigenetic relevance in cell differentiation, which is essential for the specific function(s) of complex organs, and is represented in a diverse molecular regulation of genes and gene pathways. The results also emphasize the paternal contribution to adaptive processes.

**Electronic supplementary material:**

The online version of this article (10.1007/s00335-020-09832-6) contains supplementary material, which is available to authorized users.

## Introduction

Although each cell of a mammal contains the same genotype in its nucleus, there are ~ 300 cell types with specific phenotypes (Bird 2002; Lister et al. 2009; Varley et al. 2013). Epigenetic modifications are one of the underlying molecular mechanisms contributing to this genetic plasticity (Jablonka and Raz 2009). In response to changing environments, such as temperature increase, epigenetic modifications can flexibly change and regulate gene expression (Bird 2002; Szyf 2009). Furthermore, patterns of DNA methylation, the most stable epigenetic modification, can be transmitted to subsequent generations, preparing the offspring for environments experienced by their parents and thus likely to be encountered by the offspring too (Anway et al. 2008; Carone et al. 2010; Dolinoy et al. 2006). Mainly methylation changes in promoter regions of the genome have been associated with regulation in gene expression (Deaton & Bird 2011; He et al. 2011; Lister et al. 2009). However, methylation within the gene body (intragenic) can also have a gene regulatory impact as they can have both gene silencing and activating function (Hahn et al. 2011; Jjingo et al. 2012). DNA methylation also plays a crucial role in cell differentiation, wherefore different cell types vary in their methylation patterns and have different gene expression patterns.

CpG islands (CGIs) are discrete CpG-rich DNA sequences associated with transcription start sites and often have promoter-like features. The ambiguous role of CGI methylation in cell differentiation was analyzed in cells of the mouse hematopoietic lineage where large differences in the expression of several genes studied were accompanied by surprisingly few differences in DNA methylation pattern of their promoters (Deaton et al. 2011). However, many DNA methylation differences were detected between hematopoietic cells and cells of a distantly related tissue, the brain. Interestingly, some CpG islands that are located close to tissue-specifically expressed genes may be demethylated yet their associated genes are silent. Examples of such genes are the tissue-specifically expressed human-globin (Bird et al. 1987) and alpha2(I) collagen (McKeon et al. 1982). Both have CpG islands that were unmethylated in all tissues studied, regardless of their expression. In addition, a small proportion of all CpG islands becomes methylated during development, rendering the associated promoter permanently deactivated (Bird 2002).

Besides promoter methylation, enhancer methylation is also involved in tissue-specific gene expression. A study on DNA methylation patterns in conventional (*T*_conv_; CD4^+^) and regulatory (*T*^reg^; CD4^+^CD25^+^) human T cells showed the methylation status of enhancer regions to be cell-type specific, suggesting that enhancers are contributing to regulate the cell´s fate (Schmidl et al. 2009).

Regarding studies in mammals, most studies so far have focused on lab strains, which are usually highly inbred and have been kept under artificial environments for many generations (Carone et al. 2010; Dolinoy et al. 2006; Gapp et al. 2017). However, epigenetic responses in wild mammals may completely differ from that in lab strains as wild mammals are constantly exposed to changing environments (e.g. seasonal vegetation and temperature). And although research on inbreed strains will likely miss the functional responses of a genetically heterogeneous species to environmental alterations (e.g. climate change) (Kilvitis et al. 2014; Penuelas et al. 2013), studies in wild mammals are still surprisingly rare (Kilvitis et al. 2014).

The few epigenetic studies on wild species exposed to ecologically relevant impacts such as changes in ambient temperature and/or food quality have been carried out in plants (Herrera and Bazaga 2011; Richards et al. 2017), insects (Kucharski et al. 2008), fish (Varriale and Bernardi 2006) and rodents (Weyrich et al. 2018, 2019, 2016b), and some studies investigated epigenomics in social mammals (Guerrero et al. 2020). Other studies dealt with vertebrates in which the sex of the offspring is determined by a temperature-dependent process, such as in turtles, crocodiles and some fish (Navarro-Martin et al. 2011; Valenzuela and Lance 2004). Whether epigenetic mechanisms also affect fitness is still strongly debated (Schrey et al. 2012).

In previous studies we detected DNA methylation changes in naïve wild guinea pig sons sired by fathers that had been exposed to changes of two different environmental factors, an increase in ambient temperature (Weyrich et al. 2016a, 2016b) and diet composition (Weyrich et al. 2018). We further compared the epigenetic effects of both environmental factors and found both an environmental factor-specific response, demonstrated in the differential methylation of factor-specific response genes and gene pathways, and a general response, demonstrated by methylation changes in genes and pathways that overlapped between the responses to both factors (Weyrich et al. 2019).

Although a mammal reacts as a unit to environmental changes, the epigenetic response of different tissues to such a change are likely to vary. Yet, whether this is the case, or if the response is rather general and largely independent from the organ has not been investigated, neither in model species nor in wild mammals. To address this gap, we used the wild guinea pig *Cavia aperea* as model species. Wild guinea pigs occur over almost all of South-America; they are generalists living at high and low altitudes at different vegetation zones (Asher et al. 2008; Dunnum et al. 2015).

We focused on the liver as the main metabolic and thermoregulation organ and on the testes, because methylation changes there have a potentially large impact, as they can be transmitted to the next generation via the parental germ line. Both organs were taken simultaneously, at seven days of age of the male offspring, and are thus directly comparable.

**Fig. 1:**
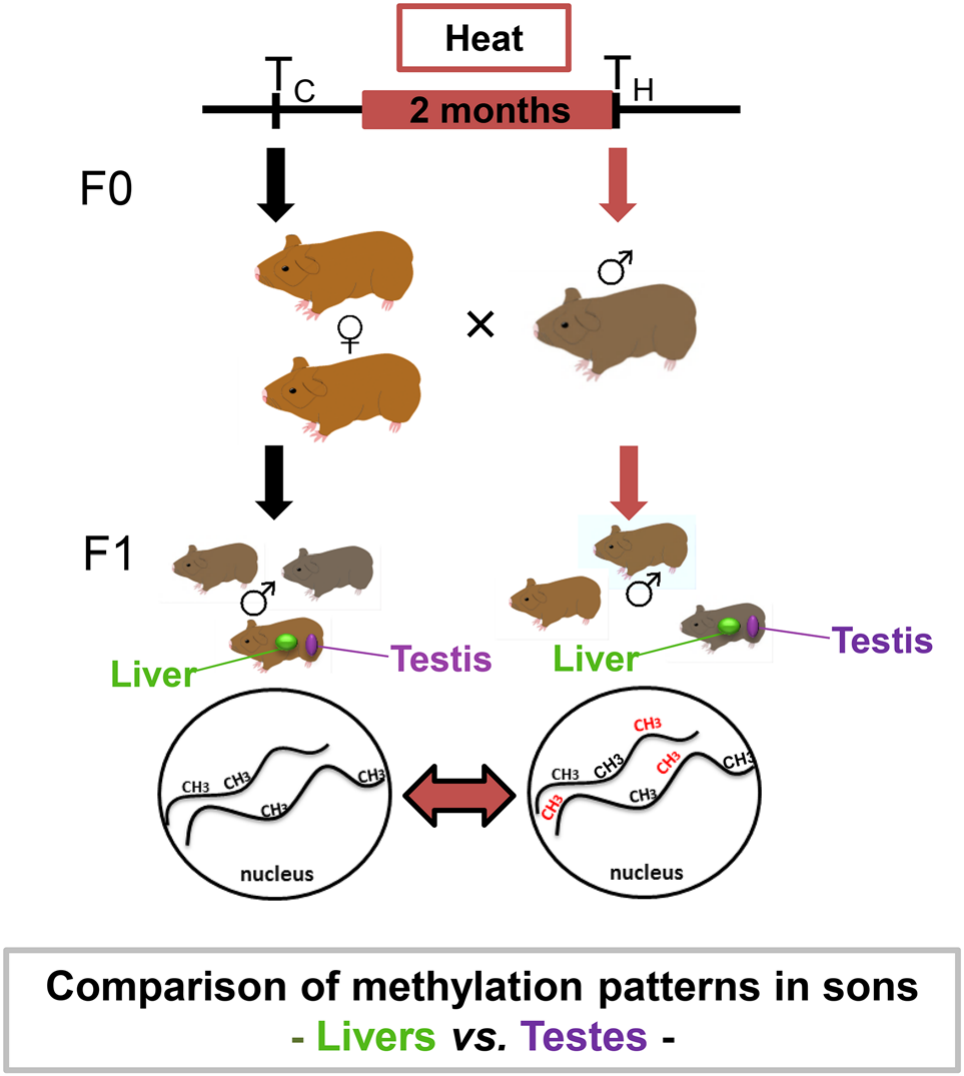
Experimental set-up and study aim. Male wild guinea pigs (*n* = 5) were exposed to an increase in temperature (H, red bar). Each male mated with the two same females before (1st Mating, *T*_C_) and after the period of exposure (2nd Mating, *T*_H_). Sons sired before the father's exposure to environmental change represent the control groups (F1L_C_ for “liver control” and F1T_C_ for “testis control”, respectively), sons sired afterwards represent the heat (F1L_H_ and F1T_H_) group. We then analysed DNA methylation patterns before and after the fathers’ treatment to identify epigenetic inheritance. In the current study, we aimed to compare genes and gene pathways of the two organs by comparing the epigenetic responses of livers with that of testes

The experimental set-up (Fig. 1) consisted of a group of five adult male guinea pigs of the same age, whose ambient temperature had been experimentally increased by 10 °C for two months and who had mated with the same two females prior and after the exposure, siring a group of sons from each mating.

This unique sampling and experimental set-up allowed us to test the three following hypotheses (with the Null-hypothesis being ‘no changes in methylation pattern’):

**H1** Organs respond to an increase in temperature with tissue-independent *general* epigenetic modifications. Under this hypothesis changes in methylation patterns should be similar in both organs and should comprise the same genes/gene pathways.

**H2** Organs respond to an increase in temperature with tissue-*specific* epigenetic modifications. Under this hypothesis, changes in methylation patterns should be different in both organs and should comprise different genes/gene pathways.

**H3** A combination of H1 and H2. Temperature increase causes both a tissue-independent *general* and a tissue-*specific* response. Under this hypothesis changes in methylation patterns should involve both shared and different genes/gene pathways.

To test these hypotheses, we identified DMRs in liver and testis of the naive son groups sired before (control) and after (heat) paternal exposure to temperature increase, to investigate both the methylation patterns that had changed specific to each organ (and thus would be indicative of a tissue-specific response) and the ones that involved the same genes and gene pathways in both organs (indicative for a general response).

## Materials and methods

### Animal housing and experimental procedures

The experiment was performed at the IZW field station in Niederfinow, Germany and was previously described in detail (Weyrich et al. 2018, 2019, 2016b). Animal husbandry and all experimental procedures were approved of by the German Committee of Animal Welfare in Research (Permit No. V3-2347–35-2011). Adult male wild guinea pigs (F0 fathers *n* = 5, labelled F–J) were kept on heating plates at 30 °C, a 10 °C increase in ambient temperature for a duration of 62 days, which reflects the timespan of a full cycle of spermatogenesis in guinea pigs (Hingst and Blottner 1995; Holt 1977). Each male was mated twice with two females: the first mating before (*T*_C_) and the second mating after the increase in temperature (*T*_H_). Livers (L) and testes (*T*) of F1 sons (F1) were harvested at day 7 of age. Liver DNA of the sons sired before (= control; F1L_C_*n* = 16) and after the heat treatment (F1L_H_*n* = 18) were sequenced via Reduced Representation Bisulfite Sequencing (RRBS; (Meissner et al. 2005)], respectively, and methylation results were grouped by fathers for further analysis (Weyrich et al. 2018, 2016b). The DNAs from testes before (= control; F1T_C_*n* = 16) and after heat treatment (F1T_H_*n* = 18) were pooled by father (F0 fathers *n* = 5, labelled F–J) before their methylation patterns were analysed via RRBS. Reads were mapped against an inhouse-generated *C. aperea* reference sequence (Weyrich et al. 2014) using BISMARK MAPPER (Krueger and Andrews 2011) (v.0.7). Genome annotations were performed as described earlier (Weyrich et al. 2014).

### DNA methylation ratio analysis

The methylation ratio of each cytosine position was calculated as the number of reads mapping to this position and carrying a *C*, divided by the number of reads carrying either *C* or *T* at this position.

Methylation ratio was calculated by the equation:

$$\frac{C}{C+T}=methylation \,ratio \, per \,specific\, mC \, site$$

Because non-methylated Cs were deaminated to *T* by bisulfite, this equation translates to

$$\frac{mC}{mC+C}=methylation \, ratio \, of \, one \,  specific \, mC \, site$$

To determine the methylation ratio we compared methylation states of sons sired by fathers before and after heat treatment (F1L_C_*vs.* F1L_H_ and F1T_C_*vs.* F1T_H_).

### Identification and comparison of differentially methylated regions (DMRs)

We clustered mCpGs to identify differentially methylated regions (DMRs) using the software methpipe (Song et al. 2013) allowing a maximum distance of 100 bp between two CpGs as has been previously described in detail (Weyrich et al. 2019, 2016b). The software uses the Fisher's exact test to estimate the differentially methylated CpG sites between samples. For DMR calculation the input data set was generated (1) using only CpG positions with read coverage in all samples and (2) using methylation ratios with a coverage of at least 5 × per CpG position in each sample. This approach generated a conservative data set, strongly restricting the number of CpG sites.

DMRs were calculated by pairwise comparisons between ‘control’ sons (F1L_C_ and F1T_C_) and their corresponding ‘heat’ sons using either DNA derived from liver (F1L_H_) or from testis (F1T_H_) (F1L_C_*vs.* F1L_H_ and F1T_C_*vs.* F1T_H_), whereby sons were grouped according to their fathers (F–J).

Identification of DMR congruence between at least four father groups was performed by using BEDtools [v. 2.15.0; intersect and multiinter; (Quinlan and Hall 2010)]. DMRs were regarded as’annotated’ when they overlapped with either gene coding sequences (CDS), promoters, or CpG islands (CGIs).

Shuffling tests were performed on methylation ratios of F1L_C_*vs.* F1L_H_ (‘liver’) and methylation ratios of F1T_C_*vs.* F1T_H_ (‘testis’), respectively. Here, the order of the methylation ratios was permuted per position, 100 times per son-father group. Each DMR calculation (100 times) resulted in less than two (potentially false positive) DMRs.

After comparison of DNA methylation patterns in ‘liver’ (F1L_C_*vs.* F1L_H_) and ’testes’ (F1T_C_*vs.* F1T_H_), we selected for DMRs located in either CpG islands, promoter regions and/or CDS (henceforth called ‘annotated DMRs’). DMRs overlapping with promoters or coding sequences (CDS) of genes were separated into three groups: (1) genes with DMRs in livers only (F1L_C_*vs.* F1L_H_), (2) genes with DMRs in testes only (F1T_C_*vs.* F1T_H_) and (3) genes whose methylation patterns had changed in both organs (by comparing gene lists resulting from (1) and (2)).

### Gene pathways assessment of differentially methylated genes

Genes per group (see above) that overlapped with at least one DMR were submitted to the web-based String database (https://string-db.org/; Version 11.0; (Snel et al. 2000; Szklarczyk et al. 2017). String combines known and predicted protein–protein association’s data such as direct (physical) interactions, as well as indirect (functional) interactions, as long as both are specific and biologically meaningful (Snel et al. 2000). Based on a stringency score (which lies between zero and one) String will identify the number of interactions and display them (higher score: less interactions, lower score: more interactions), accompanied with reassessments of the significance p values. Here, we assume that differentially methylated genes will also differentially regulate gene expression, affecting the downstream protein expression. Thus, we submitted the genes to String to identify physiological pathways that might be regulated via DNA methylation in each of the groups.

## Results

### Total numbers of differentially methylated regions in sons

**Fig. 2:**
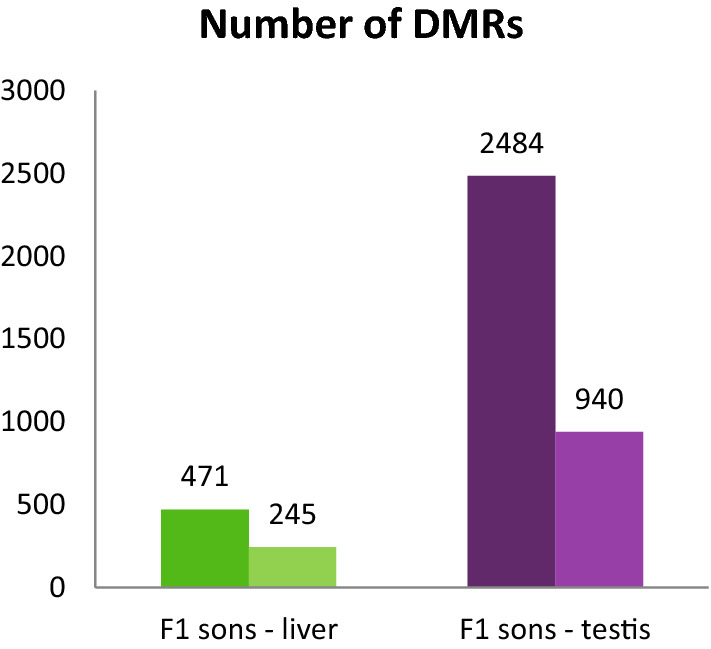
Total number and number of annotated differentially methylated regions (DMRs) in livers (green bars) and testis (violet bars) of sons sired before and after paternal heat exposure. Total number of DMRs (dark coloured) and number of annotated DMRs (light coloured) calculated between control sons and sons sired by fathers exposed to prolonged temperature increase. DMRs were regarded as ‘annotated’ when they overlapped with either gene coding sequences (CDS), promoters, or CpG islands (CGIs)

To test hypotheses H1 to H3 we compared the RRBS data to search for DNA regions with changes in their methylation patterns (differentially methylated regions; DMRs) of F1 sons in livers (F1L) and testes (F1T) after heat exposure (‘livers’: F1L_C_*vs.* F1L_H_ and ‘testes’: F1T_C_*vs.* F1T_H_). In both organs, we detected DMRs (Fig. 2). Out of the 471 DMRs detected in the liver, 245 were located in annotated regions, and out of the 2484 DMRs found in testes, 940 were located in annotated regions.

For the comparison of organ responses, we focused on DMRs within genes, including both promoter and coding sequences (CDS), which were observed in response to paternal heat exposure, either in the sons` liver or in their testes.

### Tissue-specific epigenetic response

**Fig. 3:**
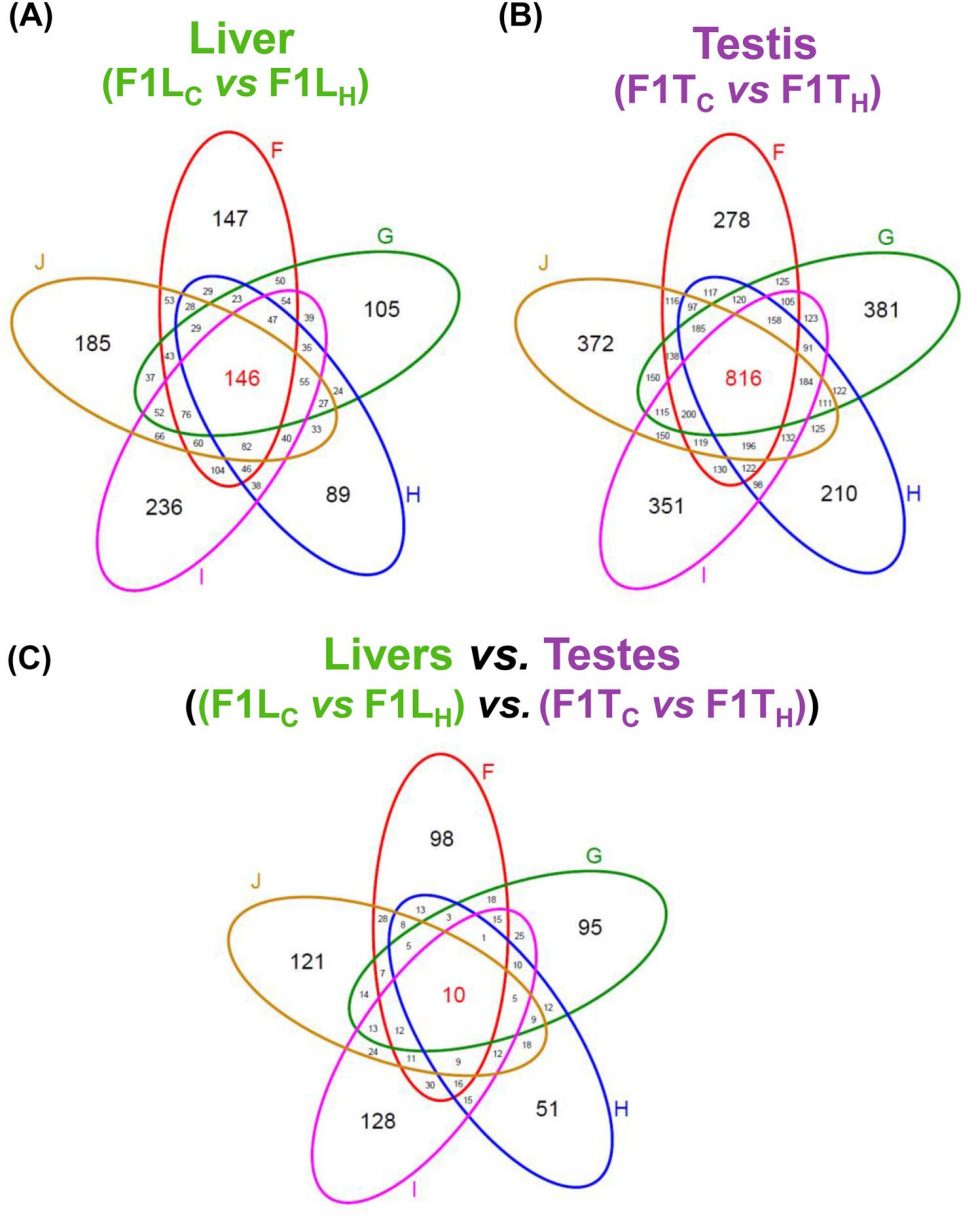
Venn diagram of annotated differentially methylated regions in F1 sons sired prior to and after paternal heat exposure. Number of annotated regions (CGIs, gene coding regions, promoters) where DMRs were detected in **a** livers of F1 sons (F1L_C_*vs*. F1L_H_), **b** testes of F1 sons (F1T_C_ vs. F1T_H_) and **c** both organs of F1 sons ((F1L_C_*vs*. F1L_H_) *vs.* (F1T_C_ vs. F1T_H_)) grouped according to their fathers (F–J)

Pairwise comparison of DMRs identified in sons grouped by father (F–J) revealed overlapping and distinctly annotated DMRs. We aimed to identify all epigenetically affected pathways, irrespectively of genes being activated or inhibited. For this reason we combined *hypo*methylated and *hyper*methylated promoters/genes in our analysis (Fig. 3).

**Fig. 4:**
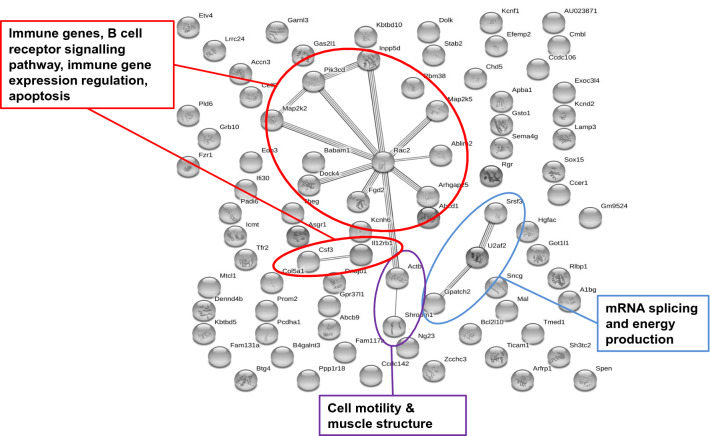
String network of genes from annotated DMRs in F1 sons sired prior to and after paternal heat exposure in ‘liver’ of all four of five father-sorted son groups. Grey dots represent single proteins encoded by differentially methylated genes. The connections between grey dots indicate protein–protein interaction. The main metabolic pathways identified are labelled by coloured circles [Figure modified from (Weyrich et al. 2019)]

**Fig. 5:**
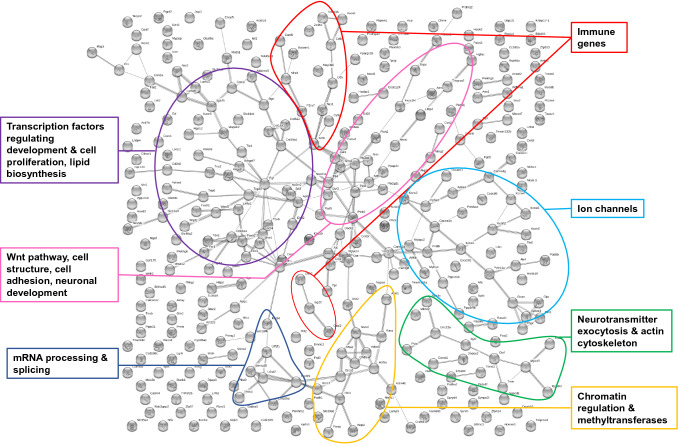
String network of genes from annotated DMRs in F1 sons ‘testes’ of all five father-sorted son groups sired before and after paternal heat exposure (F1T_C_*vs*. F1T_H_). Grey dots represent single proteins encoded by differentially methylated gene. The connections between grey dots indicate protein–protein interaction. The main metabolic pathways identified are labelled by coloured circles

For gene-network analysis, we incorporated annotated DMRs overlapping with at least one protein-coding gene that were present in at least four of the five father-sorted son groups for F1L_C_*vs.* F1L_H_ in ‘liver’ (*n*_genes_ = 84; Fig. 4; Table S1) and in all five father-sorted son groups for F1T_C_*vs.* F1T_H_ in ‘testes’ (*n*_genes_ = 322; Fig. 5; Table S2). Genes were submitted to the String database for gene network identification.

### Identification of epigenetically affected pathways in liver after paternal heat exposure

We extracted DNA from livers of sons sired after paternal heat exposure (F1L_C_*n* = 16; F1L_H_*n* = 18) and investigated each individual’s nucleotide-specific methylation pattern by RRBS sequencing. This approach resulted in individual-specific DMRs. We focused on DMRs detected in at least four father-sorted son groups to gain a more general response to paternal heat exposure within this very detailed dataset. In F1L_H_ sons we had identified 98 DMR-linked genes (Weyrich et al. 2018), of which 84 were also recognized by the String database, applying the *Mus musculus* database as a reference for gene function data. We increased the stringency interaction score from its default setting of 0.4 to 0.5. Using a higher stringency score leads to a reduction in the number of interactions detected. The interactions that are still detected, however, have a stronger backup by the available data that the String database uses for pathway detection. String network analysis displayed significantly more interactions than expected by chance (*n*_obs_ = 15, *n*_exp_ = 6, *p* = 0.0029). For the liver DMRs (F1L_C_*vs*. F1L_H_), the String network identified genes in pathways with immune function, in B cell receptor signaling, in immune genes expression, in apoptosis as well as in cell structure formation, in RNA splicing and energy production (Fig. 4).

### Identification of epigenetically affected pathways in testis after paternal heat exposure

In testis, 322 out of 361 genes with DMRs identified were recognized by the String database, again using gene function data of *Mus musculus* as a reference (Fig. 5). Even though we had increased the stringency interaction detection score from 0.4 to 0.5, the network analysis resulted in significantly more interactions than would be expected in a random data set of similar size (*n*_obs_ = 255, *n*_exp_ = 160, *p* = 2.49e–12). This indicated that the proteins are at least partially biologically connected as a group. After extraction, the DNAs of sons’ testes had been equally molarly pooled according to father to reduce sequencing costs and to facilitate data handling. However, this cost saving approach is traded for the loss of individual-specific DMRs analysis. We, therefore, chose the most conservative approach focussing on DMRs, which were detected in testes of all five father-sorted son groups. The network identified genes in pathways with main metabolic functions such as cell proliferation, *Wnt* signaling pathway, immune system, locomotory, energy synthesis, as well as transcriptional gene regulation by transcription factors, RNA splicing, methyltransferases and chromatin structure.

### Annotated differentially methylated regions shared between both tissues of sons in response to paternal heat exposure

We detected 19 annotated DMRs overlapping genes (CDS and promoter regions) that were shared between livers and testes in all five father-sorted son groups in response to prolonged paternal heat exposure. Of these annotated DMRs, 12 were located in CDS and seven in promoter regions (Table 1). No pathway-like connections were found when submitting those 19 genes to the String database. According to gene ontology terms (GO; Table 1) those 19 genes are involved in locomotory behaviour, embryonic development*,* cell proliferation, immune system, DNA repair and chromatin organisation, energy balance, homeostasis and ion transport, transcription regulation, apoptosis, spermatogenesis and exocytosis.

**Table 1:** In total, 19 genes (CDS and promoter regions) with DMRs were shared among all five father-sorted son groups sired before and after paternal heat exposure between their ‘livers’ and ‘testes’

Gene name (ensembl ID)	Full gene name	Regulatory region	Gene ontology (GO) term
*Apba1* (ENSCPOG00000003239)	Amyloid Beta Precursor Protein Binding Family A Member 1	CDS	Locomotory behavior, in utero embryonic development, intracellular protein transport, glutamate secretion, gamma-aminobutyric acid secretion, multicellular organism growth, synaptic transmission, regulation of gene expression
*Babam1* (ENSCPOG00000006927)	BRISC and BRCA1 A Complex Member 1	CDS	Positive regulation of DNA repair, chromatin organization
*C9orf142* (ENSCPOG00000019861)	PAXX Non-Homologous End Joining Factor	CDS	Cellular response to DNA damage stimulus, double-strand break repair via nonhomologous end joining
*Chd5* (ENSCPOG00000025080)	Chromodomain Helicase DNA-Binding Protein 5	Promoter	Regulation of transcription, DNA-templated, metabolic process, ATP catabolic process
*Cmbl* (ENSCPOG00000002024)	Carboxymethylenebutenolidase Homolog	CDS	Metabolic process, hydrolase activity
*Exoc3l4* (ENSCPOG00000010016)	Exocyst Complex Component 3 Like 4	CDS	Exocytosis
*Fgd2* (ENSCPOG00000001215)	FYVE, RhoGEF And PH Domain Containing 2	CDS	Regulation of Rho GTPase activity, Rho protein signal transduction
*Fzr1* (ENSCPOG00000019570)	Fizzy And Cell Division Cycle 20 Related 1	Promoter	Positive regulation of cell proliferation, activation of anaphase-promoting complex activity, negative regulation of cell aging, regulation of meiosis, lens fiber cell differentiation, anaphase-promoting complex-dependent proteasomal ubiquitin-dependent protein catabolic process, positive regulation of protein catabolic process, G2 DNA damage checkpoint
*Icmt* (ENSCPOG00000025334)	Isoprenylcysteine Carboxyl Methyltransferase	Promoter	Protein methylation, C-terminal protein methylation, in utero embryonic development, regulation of Ras protein signal transduction, positive regulation of cell proliferation, protein localization, liver development, multicellular organism growth, regulation of RNA biosynthetic process
*Kcnh6* (ENSCPOG00000001618)	Potassium Voltage-Gated Channel Subfamily H Member 6	CDS	Potassium ion transport, ion transmembrane transport, signal transduction
*Lamp3* (ENSCPOG00000015232)	Lysosomal Associated Membrane Protein 3	CDS	Adaptive immune response, regulation of autophagy
*Map2k2* (ENSCPOG00000025428)	Mitogen-Activated Protein Kinase Kinase 2	Promoter	Peptidyl-serine autophosphorylation, positive regulation of protein serine/threonine kinase activity, protein phosphorylation, positive regulation of cell motility
*Pcdha5* (ENSCPOG00000009742)	Protocadherin Alpha 5	CDS	Cell adhesion, homophilic cell adhesion
*Pik3cd* (ENSCPOG00000012950)	Phosphatidylinositol-4,5-Bisphosphate 3-Kinase Catalytic Subunit Delta	CDS	Phosphorylation, B cell activation, cell surface receptor signaling pathway, phosphatidylinositol phosphorylation, B cell homeostasis, phosphatidylinositol-mediated signaling
*Ppp1r18* (ENSCPOG00000024356)	Protein Phosphatase 1 Regulatory Subunit 18	CDS	Actin binding, phosphatase binding
*Sapcd1* (ENSCPOG00000024345)	Suppressor APC Domain Containing 1	Promoter	No information available
*Sncg* (ENSCPOG00000023463)	Synuclein Gamma	Promoter	Synapse organization, regulation of dopamine secretion, regulation of neurotransmitter secretion, adult locomotory behaviour, synaptic transmission
*Topaz1* (ENSCPOG00000001921)	Testis And Ovary Specific PAZ Domain Containing 1	Promoter	Apoptotic process, spermatocyte division, spermatid development, ncRNA transcription
*U2af2* (ENSCPOG00000001582)	U2 Small Nuclear RNA Auxiliary Factor 2	CDS	Negative regulation of mRNA splicing via spliceosomes, RNA splicing, mRNA processing

### Testing hypotheses H1–H3

Our results rejected hypotheses H1 and H2, but not H3, because changes in the two organs revealed both a *general* (tissue-independent) and a *tissue-specific* response, represented by methylation changes that are targeted both at shared and at specific gene pathways.

## Discussion

In the current study, we analysed whether different organs of F1 sons responded epigenetically similarly or differently to a prolonged exposure of their fathers to an increased ambient temperature. Analyses were carried out in liver and testis of wild guinea pig F1 sons sired prior and after paternal heat exposure. We found both, highly tissue-specific changes in methylation patterns of genes with function in the specific physiological pathways, and tissue-independent methylation changes in single specific genes. Thus, our data support hypothesis H3, the combination of both a tissue-independent *general* and a tissue-*specific* response in both organs.

These results show that paternally experienced changes of ambient temperature result in systemic epigenetic modifications in the offspring, transmitted from father to son. Studying liver and testis reflects two types of information conveyed to the subsequent generation(s): one is the metabolic response of genes in the liver coping with temperature increase (‘metabolic information’) and the other may be transmitted to subsequent generations via the testis` germ cells (‘heritable information’).

Although such “composite response” had previously been demonstrated to exist in sons after paternal exposure when the comparison was between changes of different environmental factors, e.g. epigenetic response to heat (changes in ambient temperature) *vs.* epigenetic responses to diet changes (changes in diet composition), the composite responses had been observed in the context of the same organ (liver) responding to changes of different environmental factors (Weyrich et al. 2019). A similar “composite response” is obviously mounted by different organs in sons after paternal exposure to changes of a single environmental factor (temperature). Considering that epigenetically regulated gene expression is involved in the differentiation of the ~ 300 mammalian cell types (Bird 2002; Lister et al. 2009; Varley et al. 2013), it appears to be very plausible that epigenetic mechanisms also (differentially) govern the responses of these cell types to changes in their environments.

DNA methylation changes in different functional units of organisms may provide an increased adaptability for the offspring to changes in environmental factors (e.g. temperature) by increasing the phenotypic plasticity to intrinsic and extrinsic factors and transmitting those to the offspring. In an evolutionary context, epigenetic modifications may be the intermediary between quick and short-lasting physiological responses and the resulting phenotypic diversity via very long-lasting mutational changes.

### Ecological epigenetics

In the growing field of “environmental epigenetics” the majority of studies focus on disease phenotypes after extreme exposures of inbred lab strains, cell cultures or human blood cells (Baccarelli and Bollati 2009; Bollati and Baccarelli 2010). In contrast, in our experimental set-up, we exposed male wild guinea pigs to a rather moderate 10 °C increase in ambient temperature to 30 °C. This increase is within the temperature change range the animals experience in their natural habitat in South-America where they are widely distributed (Asher et al. 2008; Dunnum et al. 2015).

Studies on wild species in comparison to inbred strains or animals kept for generations under artificial conditions can yield very different responses to the same environmental changes their inbred relatives had been exposed to. This has been shown in plants, where the two plant species *Arabidopsis thaliana* and *Spartina alterniflora* had been either less or more resilient to oil pollution, respectively, than their laboratory conspecifics (Alvarez et al. 2018). Differences in resilience were accompanied with differences in transcription and xenobiotic response pathways. Those results show that ecological studies that employ epigenomics on wild species have the potential to uncover new insights into adaptation.

### Epigenetic response to temperature increase

Those ecological studies are increasing, but are still scares, but worth investigating. Temperature is a strong selection factor, influencing physiological processes (e.g. ageing, sex ratio in reptiles), behaviour (Radchuk et al. 2019) and phenotypes of living organisms (Sonna et al. 2002). In mammals, an increase in temperature has been shown to impair spermatogenesis, by the induction of apoptosis and DNA damage, leading to a reduction of sperm quality and thus reproductive fitness (Falk and Issels 2001; Pagani et al. 2007; Sharpe 2010). Studies on domestic mammals showed, that heat shock affects the transmission and reprogramming of epigenetic information in early embryonic development of rat, mice, pigs, sheep and cattle (Sun et al. 2019). However, the epigenetic response to heat exposure has not been studied in wild mammals, other than the wild guinea pig (Sun et al. 2019). But studies exists on other species, including plants, corals, insects (fruit fly), chicken and fish which are providing first insights.

Exposure to heat in Arabidopsis, a plant model species, resulted in transcriptional activation of repetitive elements that are epigenetically controlled (Pecinka et al. 2010). Surprisingly, these expression changes are not associated with a change in DNA methylation patterns and cause only slight changes in histone modifications. It has been supposed that the activation might be regulated by chromatin reassembly, with recovery in the same generation, but impairment in the next generation. Another interesting study shows, that the plant flowering locus, defining the onset of flowering, is epigenetically regulated during the temperature-dependent vernalisation process (Bouché et al. 2015). Coral reef species change their gene expression after an increased temperature, which is associated with a decrease in DNA methylation (Dimond and Roberts 2015).

In the fruit fly *Drosophila melanogaster*, an exposure to strong heat and osmotic stress induced phosphorylation of transcription factor 2 (dAFT-2) in pacemaker neurons, responsible for sleep regulation and locomotory activity. Furthermore, it resulted in an impaired chromatin structure by releasing ATF-2 from the heterochromatin for generations (Seong et al. 2011). In chicken an epigenetic response in the frontal hypothalamus was observed after heat exposure and reexposure within the same generation. This resulted in expression changes of the brain derived neurotropic factor (BDNF) and DNA methylation changes in its promoter region (Yossifoff et al. 2008). A greater global change in DNA methylation has been shown in a comparison between arctic and tropical fish species (Varriale and Bernardi 2006). In vertebrates, such as in turtles, crocodiles and fish species, sex determination is a temperature-dependent process (Valenzuela and Lance 2004), which is epigenetically regulated (Parrott et al. 2014). For example in European sea bass females, the gonadal aromatase (CYP19A), the enzyme that converts androgen to oestrogen is methylated in its promoter region (Navarro-Martin et al. 2011). These examples demonstrate the great influence temperature has in triggering environmentally driven response processes, which are at least partly regulated by epigenetic mechanisms. It is noteworthy that epigenetic mechanisms differ among different species (e.g. invertebrates *vs.* vertebrates) (He et al. 2011). Therefore a direct comparison among them is limited, as well as a direct comparison with our data. For example, the fruit fly *Drosophila melanogaster* shows low levels of DNA methylation (Weyrich et al. 2008), but possesses a complex machinery of histone modifications (Hennig and Weyrich 2013). Plants, in contrast to mammals have in addition to CpG methylation, also high levels of CHG and CHH methylation (in which H stands C, A or T, but not G). Fishes and amphibians show in general a higher degree of DNA methylation than mammals and birds (Jabbari et al. 1997).

### Tissue-specific response to temperature increase

In the current study we demonstrate that epigenetic responses are not only cue-specific (Weyrich et al. 2019), but are also to a high proportion tissue/organ–specific, with some response overlap between the organs.

### Tissue-specific response in liver (‘metabolic information’)

The liver is the main metabolic and thermoregulation organ (‘metabolic information’). It stores energy in form of glycogen, whose hydrolysis to glucose subsequently generates the energy required for a systemic response. In our experiment, the exposure of fathers to increased ambient temperature triggered a specific epigenetic response in the livers of F1 sons, measured as changes in methylation patterns. These changes were seen in genes belonging to pathways responsible for immune function, B cell receptor signalling, immune gene expression, apoptosis, as well as in cell structure, RNA splicing and energy production (Fig. 4). Most of those genes are relevant for the immune system, indicating a response that is directed to maintain or to improve the animals’ health status. A temperature increase may lead to apoptosis and DNA damage (Falk and Issels 2001; Pagani et al. 2007; Sharpe 2010), and also affects nutritional, physiological and reproductive functions and potentially impedes spermatogenic activity, rendering the activation of immune system components a very plausible epigenetic response.

### Whole liver samples and cell differentiation

DNA methylation is a mechanism regulating cell differentiation, wherefore DNA methylation patterns differ greatly among cell types and tissues, accompanied with divers gene expression patterns (Deaton et al. 2011). The differentiation of cells into functionally different cell types is mainly regulated by methylation, wherefore different cell types have different methylomes (Khavari et al. 2010; Michalowsky and Jones 1989). A limitation of our study is the use of liver and testis homogenates as DNA source. Both organs do not consist of just a single cell type but of a tightly intertwined community of cell types that all may differ in their epigenomes. Thus, the results we obtained per organ only reflect the average of methylation changes across the cell types analysed in both organs. For the question we were addressing, namely whether there are differences in the average epigenome of two different organs in response to the same external input, such differences in the epigenomes across different cell types are negligible. However, to avoid biased results by over or underrepresentation of certain cell types we homogenized the entire organ, reducing our prior assumptions to just the one that the cellular composition of the livers was very similar across individuals. To further strengthen our interpretations we constrained our analyses to differentially methylated genomic regions that were present with high reproducibility among all fathers-son groups analysed. If one of the responses would have resulted in a shift of cell type ratio of the organs, this shift would have occurred in all sons sired (after paternal exposure), which may also be a response to the environmental factor. Furthermore, we validated DMRs occurrence using a shuffle test.

### Tissue-specific response in testis (‘heritable information’)

Testes are the male reproductive organs producing sperm germ cells by spermatogenesis. Epi/genetic information carried by the sperm’s DNA can be transmitted to subsequent generations, wherefore the testes are the most important organ for male inheritance (‘heritable information’). In response to paternal heat exposure, genes differentially methylated in the F1 sons belonged to pathways with main metabolic functions such as cell proliferation, *Wnt* signaling pathway, immune system, locomotory, energy synthesis, and genes important for sperm vitality. Furthermore genes were differentially methylated with importance in transcriptional gene regulation such as transcription factors, RNA splicing, methyltransferases and chromatin structure. For example, the *Wnt* signalling pathway reacts to outer stimuli by its *Wnt* (*Wingless*) and *Integrator Complex Subunit 2* (*Int-2*) signal proteins (Logan and Nusse 2004), which transmit the information to the organs, inducing metabolic processes, which stabilize or regain homeostasis (Tortora and Derrickson 2012).

### Differentiation of male germ cells & early testis development stage

Testes of the seven-days old animals incorporate germ cells in a differentiated and undifferentiated stage (Kubo et al. 2015). Because DNA methylation is involved in cell differentiation processes (as described above), some changes in methylation patterns occur due to differentiation of cell types. However, we have measured DMRs, regions that had been differentially methylated both in the control group of sons (sired prior paternal exposure) and in the son group sired after paternal exposure. In both cases the testes contained cells that were still in the process of differentiation. However, the ongoing cell differentiation may at least partially explain the ~ 5 times higher number of DMRs (2,484 DMRs) found in testis of sons sired before and after the heat treatment of fathers (F1T_C_*vs.* F1T_H_), while liver samples of identical animals had only 471 DMRs (Fig. 2).

### Tissue-independent general response

The *general* response we detected, was independent of the tissue studied, consisting of genes with DMRs observed in both organs (Table 1). These genes are components of pathways involved in locomotory behaviour (*Apba1, Sncg, Ppp1r18*), embryonic development (*Icmt, Ppp1r18),* cell proliferation (*Fzr1, Icmt, Ppp1r18)*, immune system (*Lamp3, Map2k2, Pik3cd, Pcdha5*), DNA repair (*Babam1, C9orf142*) and chromatin organisation *(Babam1, C9orf142)*, energy balance (*Fgd2, Chd5, Cmbl)*, homeostasis (*Pik3cd)* and ion transport (*Kcnh6)*, transcription regulation (*Chd5, U2af2)* and apoptosis (*Topaz1),* and in spermatogenesis (*Topaz1)* and exocytosis (*Exoc3l4*). The genes detected are important for successful embryonic development and energy balance, and thus are expected to be responding in both organs as these are elementary functions of testis and liver. It is noteworthy, that DMRs at genes can be *hypo* or *hyper*methylated and as such they might be differentially expressed in both organs.

Whether such epigenetic responses of the offspring to paternal exposure to temperature increase is a species-specific phenomenon or is a general response pattern that can be found across rodents or even mammals (as we hypothesize) remains to be investigated. So far, to the best of our knowledge, epigenetic studies on other wild mammals exposed to a prolonged increase in ambient temperature were not carried out yet (see above).

### Parental age-effect

A major goal of our study was to study identical F0 males before and after exposure, to ensure identical genomes. Here, we like to point out that even though the experimental set-up was designed to decrease the time-span in between both mating to a minimal, the first and second mating were about 7.5 months apart. Therefore, F0 males grew ~ 7.5 months older until the second mating; giving the chance that DNA methylation may have changed in an age-dependent manner (Fraga et al. 2005). This is a point that needs to be addressed in future research.

### Epigenetic regulation of gene expression

As pointed out above (“Material and Methods” section), it is noteworthy that the network-analysis within the current study is based on the assumption that the DMRs we found to be located in gene regions effect the expression of the respective gene. To address this experimentally, additional transcriptome (e.g. from RNA-Seq) data would need to be analysed, and differentially expressed genes would then need to be compared with genes associated with DMRs. Therefore, a limitation of the current study is that the causal relationship of the DMR and gene expression change is not known, but potentially possible due to genome location.

## Conclusions

Our data support the hypothesis that paternal exposure to a prolonged increase in ambient temperature leads to an inherited differential response in the two organs studied in the F1 sons. Livers and testes of the sons elicit both a tissue-independent *general* and a tissue-*specific* response, whereby the specifically regulated proportion of genes was much higher than the proportion of regulated genes shared between the two organs. These specifically epigenetically regulated genes we refer to as ‘metabolic information’ in liver and as ‘heritable information’ in testis.

## Electronic supplementary material

Below is the link to the electronic supplementary material.

Supplementary file 1 (DOCX 62 kb)
