# Supplementary material for: Tissue-specific epigenetic inheritance after paternal heat exposure in male wild guinea pigs
Source: Mamm Genome. 2020 Apr 13;31(5):157–69. doi: 10.1007/s00335-020-09832-6 (PMC7369130; doi:10.1007/s00335-020-09832-6)
Supplement: Supplementary file 1 — (DOCX 62 kb) [file 335_2020_9832_MOESM1_ESM.docx]

**Supplementary Materials**

Next generation sequencing data are available online (<http://www.ncbi.nim.nih.gov/sra>) (Appendix A). Table S1: Annotated DMRs in ‘liver’ present in at least four of five father-sorted son groups (F1L_CH_ *vs.* F1L_H_) with at least one annotated gene of protein-coding genes present (applied for Fig. 4) (Appendix B), Table S2: List of annotated DMRs in ‘testis’ present in all father-sorted son groups (F1T_CH_ *vs.* F1T_H_) with at least one annotated gene of protein-coding genes (applied for Fig. 5).

**Supplementary Material**

**Table S1:** Annotated DMRs in ‘liver’ present in at least four of five father-sorted son groups (F1L_C_ *vs.* F1L_H_) with at least one annotated gene of protein-coding genes present (applied for Fig. 4)

| **Id** | **Gene name** | **Annotation type** | **No. of fathers-son groups** |
| --- | --- | --- | --- |
| ENSCPOG00000023463_tss | SNCG | tss | 4 |
| ENSCPOG00000026310_promoter | unknown gene | promoter | 5 |
| ENSCPOG00000025943_promoter | ARHGAP25 | promoter | 4 |
| ENSCPOG00000025820_promoter | IL12RB1 | promoter | 4 |
| ENSCPOG00000025541_promoter | GPR37L1 | promoter | 4 |
| ENSCPOG00000025428_promoter | MAP2K2 | promoter | 4 |
| ENSCPOG00000025389_promoter | BCL2L10 | promoter | 5 |
| ENSCPOG00000025334_promoter | ICMT | promoter | 4 |
| ENSCPOG00000024373_promoter | TTC31 | promoter | 4 |
| ENSCPOG00000024356_promoter | PPP1R18 | promoter | 5 |
| ENSCPOG00000023502_promoter | Ccer1 | promoter | 5 |
| ENSCPOG00000023391_promoter | COL5A1 | promoter | 4 |
| ENSCPOG00000022128_promoter | MAL | promoter | 4 |
| ENSCPOG00000021900_promoter | Rac2 | promoter | 5 |
| ENSCPOG00000020508_promoter | C5orf45 | promoter | 5 |
| ENSCPOG00000019570_promoter | FZR1 | promoter | 4 |
| ENSCPOG00000013933_promoter | SOGA2 | promoter | 4 |
| ENSCPOG00000012427_promoter | PLD6 | promoter | 5 |
| ENSCPOG00000011847_promoter | KCND2 | promoter | 4 |
| ENSCPOG00000009704_promoter | ABLIM2 | promoter | 4 |
| ENSCPOG00000006486_promoter | TICAM1 | promoter | 4 |
| ENSCPOG00000003083_promoter | unknown gene | promoter | 4 |
| ENSCPOG00000002302_promoter | TMED1 | promoter | 4 |
| ENSCPOG00000001582_promoter | U2AF2 | promoter | 5 |
| ENSCPOG00000023463_start_codon | SNCG | codon | 4 |
| ENSCPOG00000026460_CDS | C2orf54 | CDS | 4 |
| ENSCPOG00000025080_CDS | CHD5 | CDS | 4 |
| ENSCPOG00000024746_CDS | GOT1L1 | CDS | 4 |
| ENSCPOG00000024356_CDS | PPP1R18 | CDS | 5 |
| ENSCPOG00000024345_CDS | SAPCD1 | CDS | 4 |
| ENSCPOG00000024187_CDS | Padi6 | CDS | 4 |
| ENSCPOG00000023974_CDS | ZCCHC3 | CDS | 4 |
| ENSCPOG00000023599_CDS | HGFAC | CDS | 4 |
| ENSCPOG00000023486_CDS | CCDC142 | CDS | 4 |
| ENSCPOG00000023463_CDS | SNCG | CDS | 4 |
| ENSCPOG00000023234_CDS | A1BG | CDS | 4 |
| ENSCPOG00000022556_CDS | RGR | CDS | 4 |
| ENSCPOG00000022172_CDS | ABCB9 | CDS | 4 |
| ENSCPOG00000021785_CDS | C10orf105 | CDS | 5 |
| ENSCPOG00000021071_CDS | C6orf25 | CDS | 4 |
| ENSCPOG00000020425_CDS | SHROOM1 | CDS | 4 |
| ENSCPOG00000019906_CDS | FAM117A | CDS | 4 |
| ENSCPOG00000019861_CDS | C9orf142 | CDS | 4 |
| ENSCPOG00000019765_CDS | GAS2L1 | CDS | 5 |
| ENSCPOG00000019701_CDS | ASIC3 | CDS | 5 |
| ENSCPOG00000015568_CDS | LRRC24 | CDS | 4 |
| ENSCPOG00000015432_CDS | KCNF1 | CDS | 5 |
| ENSCPOG00000015413_CDS | C6orf136 | CDS | 4 |
| ENSCPOG00000015232_CDS | LAMP3 | CDS | 4 |
| ENSCPOG00000015231_CDS | KBTBD5 | CDS | 4 |
| ENSCPOG00000015128_CDS | CSF3 | CDS | 5 |
| ENSCPOG00000014919_CDS | SH3TC2 | CDS | 5 |
| ENSCPOG00000014435_CDS | DNAJB1 | CDS | 5 |
| ENSCPOG00000014423_CDS | ARFRP1 | CDS | 4 |
| ENSCPOG00000014036_CDS | SPEN | CDS | 4 |
| ENSCPOG00000013467_CDS | Actb | CDS | 5 |
| ENSCPOG00000012950_CDS | PIK3CD | CDS | 4 |
| ENSCPOG00000012910_CDS | DENND4B | CDS | 4 |
| ENSCPOG00000012781_CDS | RBM38 | CDS | 4 |
| ENSCPOG00000012432_CDS | DOCK4 | CDS | 5 |
| ENSCPOG00000012318_CDS | GARNL3 | CDS | 4 |
| ENSCPOG00000011702_CDS | ABCD1 | CDS | 4 |
| ENSCPOG00000011364_CDS | INPP5D | CDS | 4 |
| ENSCPOG00000011162_CDS | TFR2 | CDS | 4 |
| ENSCPOG00000010952_CDS | B4GALNT3 | CDS | 5 |
| ENSCPOG00000010016_CDS | EXOC3L4 | CDS | 4 |
| ENSCPOG00000009813_CDS | ASGR1 | CDS | 4 |
| ENSCPOG00000009742_CDS | PCDHA5 | CDS | 4 |
| ENSCPOG00000009639_CDS | RLBP1 | CDS | 4 |
| ENSCPOG00000009223_CDS | GRB10 | CDS | 4 |
| ENSCPOG00000009196_CDS | FAM131A | CDS | 4 |
| ENSCPOG00000008349_CDS | THEG | CDS | 4 |
| ENSCPOG00000008083_CDS | EFEMP2 | CDS | 5 |
| ENSCPOG00000007922_CDS | C20orf85 | CDS | 4 |
| ENSCPOG00000006927_CDS | Babam1 | CDS | 4 |
| ENSCPOG00000006097_CDS | PROM2 | CDS | 4 |
| ENSCPOG00000005553_CDS | IFI30 | CDS | 4 |
| ENSCPOG00000004968_CDS | KBTBD10 | CDS | 4 |
| ENSCPOG00000004915_CDS | SOX15 | CDS | 5 |
| ENSCPOG00000004736_CDS | EDN3 | CDS | 4 |
| ENSCPOG00000004082_CDS | DOLK | CDS | 4 |
| ENSCPOG00000003966_CDS | GPATCH2 | CDS | 4 |
| ENSCPOG00000003722_CDS | SRSF3 | CDS | 4 |
| ENSCPOG00000003539_CDS | CELF3 | CDS | 4 |
| ENSCPOG00000003371_CDS | STAB2 | CDS | 4 |
| ENSCPOG00000003362_CDS | ETV4 | CDS | 4 |
| ENSCPOG00000003239_CDS | APBA1 | CDS | 4 |
| ENSCPOG00000003125_CDS | BTG4 | CDS | 4 |
| ENSCPOG00000002716_CDS | CYP21A2 | CDS | 4 |
| ENSCPOG00000002024_CDS | CMBL | CDS | 4 |
| ENSCPOG00000001921_CDS | TOPAZ1 | CDS | 4 |
| ENSCPOG00000001618_CDS | KCNH6 | CDS | 5 |
| ENSCPOG00000001215_CDS | FGD2 | CDS | 4 |
| ENSCPOG00000001014_CDS | GSTO1 | CDS | 4 |
| ENSCPOG00000000970_CDS | MAP2K5 | CDS | 4 |
| ENSCPOG00000000944_CDS | SEMA4G | CDS | 5 |
| ENSCPOG00000000682_CDS | PTHR11347_SF105 | CDS | 4 |
| ENSCPOG00000000131_CDS | CCDC106 | CDS | 5 |

CDS, coding sequence; tss, transcription start side

**Table S2:** List of annotated DMRs in ‘testis’ present in all father-sorted son groups (F1T_C_ *vs.* F1T_H_) with at least one annotated promoter and/or protein-coding gene (CDS) (applied for Fig. 5)

| **Id** | **Gene name** | **Annotation type** | **No. of son-father groups** |
| --- | --- | --- | --- |
| ENSCPOG00000000010_promoter | H6PD | CDS | 5 |
| ENSCPOG00000000038_CDS | MIDN | CDS | 5 |
| ENSCPOG00000000062_CDS | PGLYRP1 | CDS | 5 |
| ENSCPOG00000000207_CDS | TECPR2 | CDS | 5 |
| ENSCPOG00000000331_CDS | OTOF | CDS | 5 |
| ENSCPOG00000000372_CDS | PDZRN4 | CDS | 5 |
| ENSCPOG00000000387_CDS | PHKG1 | CDS | 5 |
| ENSCPOG00000000576_CDS | PLEKHG6 | CDS | 5 |
| ENSCPOG00000000591_CDS | NOTCH4 | CDS | 5 |
| ENSCPOG00000000682_CDS | PDE4B | CDS | 5 |
| ENSCPOG00000000713_CDS | TRIOBP | CDS | 5 |
| ENSCPOG00000000771_CDS | HOOK2 | CDS | 5 |
| ENSCPOG00000000787_CDS | FARSA | CDS | 5 |
| ENSCPOG00000000914_CDS | CAMK2G | CDS | 5 |
| ENSCPOG00000000959_promoter | THOC1 | CDS | 5 |
| ENSCPOG00000000966_CDS | IQCH | CDS | 5 |
| ENSCPOG00000000999_CDS | ICAM5 | CDS | 5 |
| ENSCPOG00000001024_CDS | ULK1 | CDS | 5 |
| ENSCPOG00000001038_CDS | Kcnn4 | CDS | 5 |
| ENSCPOG00000001042_CDS | TBX2 | CDS | 5 |
| ENSCPOG00000001125_CDS | PALM3 | CDS | 5 |
| ENSCPOG00000001143_CDS | LTBP2 | CDS | 5 |
| ENSCPOG00000001215_CDS | FGD2 | CDS | 5 |
| ENSCPOG00000001319_CDS | NCR1 | CDS | 5 |
| ENSCPOG00000001320_CDS | NID2 | CDS | 5 |
| ENSCPOG00000001463_CDS | EBF2 | CDS | 5 |
| ENSCPOG00000001555_CDS | FBRSL1 | CDS | 5 |
| ENSCPOG00000001556_CDS | LHFPL4 | CDS | 5 |
| ENSCPOG00000001582_CDS | U2AF2 | CDS | 5 |
| ENSCPOG00000001613_CDS | PTGIR | CDS | 5 |
| ENSCPOG00000001618_CDS | KCNH6 | CDS | 5 |
| ENSCPOG00000001717_CDS | OXT | CDS | 5 |
| ENSCPOG00000001735_CDS | SLC12A9 | CDS | 5 |
| ENSCPOG00000001808_CDS | ERF | CDS | 5 |
| ENSCPOG00000001823_CDS | SLC46A2 | CDS | 5 |
| ENSCPOG00000001858_CDS | A4GALT | CDS | 5 |
| ENSCPOG00000001881_CDS | RASIP1 | CDS | 5 |
| ENSCPOG00000001958_CDS | PPARGC1B | CDS | 5 |
| ENSCPOG00000002024_CDS | CMBL | CDS | 5 |
| ENSCPOG00000002219_CDS | CYTIP | CDS | 5 |
| ENSCPOG00000002223_CDS | MYH14 | CDS | 5 |
| ENSCPOG00000002236_CDS | SLC35E4 | CDS | 5 |
| ENSCPOG00000002349_CDS | SPRYD4 | CDS | 5 |
| ENSCPOG00000002438_CDS | GIPR | CDS | 5 |
| ENSCPOG00000002444_CDS | SSUH2 | CDS | 5 |
| ENSCPOG00000002548_CDS | SNRNP35 | CDS | 5 |
| ENSCPOG00000002818_CDS | KLHL10 | CDS | 5 |
| ENSCPOG00000003039_CDS | RASAL3 | CDS | 5 |
| ENSCPOG00000003239_CDS | APBA1 | CDS | 5 |
| ENSCPOG00000003430_CDS | MN1 | CDS | 5 |
| ENSCPOG00000003437_CDS | NA | CDS | 5 |
| ENSCPOG00000003554_CDS | PPFIA3 | CDS | 5 |
| ENSCPOG00000003645_CDS | MYO15A | CDS | 5 |
| ENSCPOG00000003728_CDS | SYNM | CDS | 5 |
| ENSCPOG00000003785_CDS | TCF23 | CDS | 5 |
| ENSCPOG00000003802_CDS | EPS8L2 | CDS | 5 |
| ENSCPOG00000003883_CDS | SLC39A8 | CDS | 5 |
| ENSCPOG00000003952_CDS | TBX3 | CDS | 5 |
| ENSCPOG00000003964_CDS | CADM4 | CDS | 5 |
| ENSCPOG00000004116_CDS | FZD5 | CDS | 5 |
| ENSCPOG00000004231_CDS | CC2D1A | CDS | 5 |
| ENSCPOG00000004320_CDS | EFCAB6 | CDS | 5 |
| ENSCPOG00000004395_CDS | LAMC3 | CDS | 5 |
| ENSCPOG00000004475_CDS | KCNA5 | CDS | 5 |
| ENSCPOG00000004477_CDS | KCNA1 | CDS | 5 |
| ENSCPOG00000004538_CDS | TMPRSS6 | CDS | 5 |
| ENSCPOG00000004565_CDS | HOMER3 | CDS | 5 |
| ENSCPOG00000004747_CDS | SH3TC1 | CDS | 5 |
| ENSCPOG00000005104_CDS | AP2A1 | CDS | 5 |
| ENSCPOG00000005141_CDS | TNS3 | CDS | 5 |
| ENSCPOG00000005171_CDS | PADI4 | CDS | 5 |
| ENSCPOG00000005334_CDS | RGS12 | CDS | 5 |
| ENSCPOG00000005411_CDS | FER1L5 | CDS | 5 |
| ENSCPOG00000005703_CDS | ESR2 | CDS | 5 |
| ENSCPOG00000005750_CDS | PCDH1 | CDS | 5 |
| ENSCPOG00000005900_CDS | ZFP57 | CDS | 5 |
| ENSCPOG00000005901_CDS | PRDM12 | CDS | 5 |
| ENSCPOG00000005904_CDS | ACTL7B | CDS | 5 |
| ENSCPOG00000006080_CDS | TIE1 | CDS | 5 |
| ENSCPOG00000006169_CDS | HOXD3 | CDS | 5 |
| ENSCPOG00000006231_CDS | GPR124 | CDS | 5 |
| ENSCPOG00000006319_CDS | PLD2 | CDS | 5 |
| ENSCPOG00000006588_CDS | GREB1 | CDS | 5 |
| ENSCPOG00000006741_CDS | AMOTL2 | CDS | 5 |
| ENSCPOG00000006785_CDS | CDKN1A | CDS | 5 |
| ENSCPOG00000006801_CDS | ITGA2B | CDS | 5 |
| ENSCPOG00000006868_CDS | PLXNB3 | CDS | 5 |
| ENSCPOG00000006916_CDS | NA | CDS | 5 |
| ENSCPOG00000006927_CDS | BABAM1 | CDS | 5 |
| ENSCPOG00000007061_CDS | PTCHD2 | CDS | 5 |
| ENSCPOG00000007086_CDS | AIM1L | CDS | 5 |
| ENSCPOG00000007417_CDS | FSTL3 | CDS | 5 |
| ENSCPOG00000007477_CDS | SBNO2 | CDS | 5 |
| ENSCPOG00000007681_CDS | NLRP3 | CDS | 5 |
| ENSCPOG00000007687_CDS | IL2RB | CDS | 5 |
| ENSCPOG00000007730_CDS | RARA | CDS | 5 |
| ENSCPOG00000007786_CDS | USH2A | CDS | 5 |
| ENSCPOG00000008026_CDS | FLT4 | CDS | 5 |
| ENSCPOG00000008161_CDS | PLEKHA4 | CDS | 5 |
| ENSCPOG00000008241_CDS | MEX3B | CDS | 5 |
| ENSCPOG00000008416_CDS | RAB3GAP2 | CDS | 5 |
| ENSCPOG00000008445_CDS | CPA2 | CDS | 5 |
| ENSCPOG00000008500_CDS | COL14A1 | CDS | 5 |
| ENSCPOG00000008577_CDS | ALPK3 | CDS | 5 |
| ENSCPOG00000008920_CDS | NA | CDS | 5 |
| ENSCPOG00000009225_CDS | PCDHGC3 | CDS | 5 |
| ENSCPOG00000009248_CDS | GNAS | CDS | 5 |
| ENSCPOG00000009344_CDS | UAP1L1 | CDS | 5 |
| ENSCPOG00000009376_CDS | PCLO | CDS | 5 |
| ENSCPOG00000009423_CDS | BHLHA15 | CDS | 5 |
| ENSCPOG00000009466_CDS | SLC27A3 | CDS | 5 |
| ENSCPOG00000009484_CDS | ERCC6 | CDS | 5 |
| ENSCPOG00000009492_CDS | LGR6 | CDS | 5 |
| ENSCPOG00000009497_CDS | GLIS1 | CDS | 5 |
| ENSCPOG00000009593_CDS | LAMA5 | CDS | 5 |
| ENSCPOG00000009738_CDS | PCDHA2 | CDS | 5 |
| ENSCPOG00000009742_CDS | PCDHA5 | CDS | 5 |
| ENSCPOG00000009899_CDS | FOXH1 | CDS | 5 |
| ENSCPOG00000009911_CDS | ADAMTS5 | CDS | 5 |
| ENSCPOG00000010016_CDS | EXOC3L4 | CDS | 5 |
| ENSCPOG00000010058_CDS | DNAH8 | CDS | 5 |
| ENSCPOG00000010080_CDS | PLEKHH3 | CDS | 5 |
| ENSCPOG00000010157_CDS | BTBD11 | CDS | 5 |
| ENSCPOG00000010200_CDS | LDHD | CDS | 5 |
| ENSCPOG00000010252_CDS | CD93 | CDS | 5 |
| ENSCPOG00000010335_CDS | TMEM132B | CDS | 5 |
| ENSCPOG00000010585_CDS | ARHGEF7 | CDS | 5 |
| ENSCPOG00000010908_CDS | TP73 | CDS | 5 |
| ENSCPOG00000010965_CDS | FOXJ1 | CDS | 5 |
| ENSCPOG00000011204_CDS | PITPNM1 | CDS | 5 |
| ENSCPOG00000011608_CDS | PPL | CDS | 5 |
| ENSCPOG00000011691_CDS | SLC2A6 | CDS | 5 |
| ENSCPOG00000011963_CDS | CACNA1I | CDS | 5 |
| ENSCPOG00000012069_CDS | PLEKHM2 | CDS | 5 |
| ENSCPOG00000012100_CDS | CDH1 | CDS | 5 |
| ENSCPOG00000012108_CDS | TRIM42 | CDS | 5 |
| ENSCPOG00000012168_CDS | MYPOP | CDS | 5 |
| ENSCPOG00000012253_CDS | PTPN21 | CDS | 5 |
| ENSCPOG00000012359_CDS | MAP3K6 | CDS | 5 |
| ENSCPOG00000012376_CDS | WASF2 | CDS | 5 |
| ENSCPOG00000012600_CDS | REXO1 | CDS | 5 |
| ENSCPOG00000012610_CDS | ZC3H4 | CDS | 5 |
| ENSCPOG00000012619_CDS | FAM184B | CDS | 5 |
| ENSCPOG00000012632_CDS | GTF2F1 | CDS | 5 |
| ENSCPOG00000012669_CDS | MBOAT7 | CDS | 5 |
| ENSCPOG00000012849_CDS | ASXL1 | CDS | 5 |
| ENSCPOG00000012864_CDS | NAPSA | CDS | 5 |
| ENSCPOG00000012916_CDS | OTUD6A | CDS | 5 |
| ENSCPOG00000012950_CDS | PIK3CD | CDS | 5 |
| ENSCPOG00000013014_CDS | PNCK | CDS | 5 |
| ENSCPOG00000013080_CDS | NGEF | CDS | 5 |
| ENSCPOG00000013124_CDS | CSRP2BP | CDS | 5 |
| ENSCPOG00000013205_CDS | PLEKHG5 | CDS | 5 |
| ENSCPOG00000013220_CDS | HMCN2 | CDS | 5 |
| ENSCPOG00000013345_CDS | DLL3 | CDS | 5 |
| ENSCPOG00000013564_CDS | RLN3 | CDS | 5 |
| ENSCPOG00000013579_CDS | PCDHGA2 | CDS | 5 |
| ENSCPOG00000013640_CDS | TMEM161A | CDS | 5 |
| ENSCPOG00000013877_CDS | DOPEY2 | CDS | 5 |
| ENSCPOG00000013914_CDS | DNAH10 | CDS | 5 |
| ENSCPOG00000013977_CDS | CELSR1 | CDS | 5 |
| ENSCPOG00000013985_CDS | WNK2 | CDS | 5 |
| ENSCPOG00000014040_CDS | DSCAML1 | CDS | 5 |
| ENSCPOG00000014208_CDS | PLBD2 | CDS | 5 |
| ENSCPOG00000014255_CDS | ALLC | CDS | 5 |
| ENSCPOG00000014293_CDS | EML3 | CDS | 5 |
| ENSCPOG00000014431_CDS | FAT2 | CDS | 5 |
| ENSCPOG00000014484_CDS | NA | CDS | 5 |
| ENSCPOG00000014627_CDS | TRIP10 | CDS | 5 |
| ENSCPOG00000014645_CDS | MYOC | CDS | 5 |
| ENSCPOG00000014755_CDS | LEFTY2 | CDS | 5 |
| ENSCPOG00000014879_CDS | MKNK2 | CDS | 5 |
| ENSCPOG00000015116_CDS | JAK3 | CDS | 5 |
| ENSCPOG00000015152_CDS | CARD9 | CDS | 5 |
| ENSCPOG00000015175_CDS | SSPO | CDS | 5 |
| ENSCPOG00000015232_CDS | LAMP3 | CDS | 5 |
| ENSCPOG00000015324_CDS | Tnnt2 | CDS | 5 |
| ENSCPOG00000015390_CDS | LRIT2 | CDS | 5 |
| ENSCPOG00000015430_CDS | ANKS1A | CDS | 5 |
| ENSCPOG00000015480_CDS | ALDH4A1 | CDS | 5 |
| ENSCPOG00000015501_CDS | CSMD1 | CDS | 5 |
| ENSCPOG00000015562_CDS | GPT | CDS | 5 |
| ENSCPOG00000015610_CDS | GLTSCR1 | CDS | 5 |
| ENSCPOG00000019364_CDS | PLCH2 | CDS | 5 |
| ENSCPOG00000019369_CDS | SYT7 | CDS | 5 |
| ENSCPOG00000019548_CDS | TNFRSF25 | CDS | 5 |
| ENSCPOG00000019861_CDS | C9orf142 | CDS | 5 |
| ENSCPOG00000019862_CDS | SHC3 | CDS | 5 |
| ENSCPOG00000019931_CDS | FIGNL2 | CDS | 5 |
| ENSCPOG00000020296_CDS | PPP1R9B | CDS | 5 |
| ENSCPOG00000020299_CDS | TNFRSF1B | CDS | 5 |
| ENSCPOG00000020688_CDS | PRPF19 | CDS | 5 |
| ENSCPOG00000020699_CDS | PNMT | CDS | 5 |
| ENSCPOG00000020962_CDS | MYBPC2 | CDS | 5 |
| ENSCPOG00000021078_CDS | NTF4 | CDS | 5 |
| ENSCPOG00000021165_CDS | TBX6 | CDS | 5 |
| ENSCPOG00000021309_CDS | TMEM44 | CDS | 5 |
| ENSCPOG00000021313_CDS | TNXB | CDS | 5 |
| ENSCPOG00000021317_CDS | RPH3A | CDS | 5 |
| ENSCPOG00000021331_CDS | KIAA1522 | CDS | 5 |
| ENSCPOG00000021341_CDS | TMPRSS9 | CDS | 5 |
| ENSCPOG00000021393_CDS | AJAP1 | CDS | 5 |
| ENSCPOG00000021486_CDS | ZC3H18 | CDS | 5 |
| ENSCPOG00000021545_CDS | NA | CDS | 5 |
| ENSCPOG00000021546_CDS | FBN3 | CDS | 5 |
| ENSCPOG00000021579_CDS | EXOC3L2 | CDS | 5 |
| ENSCPOG00000021942_CDS | HVCN1 | CDS | 5 |
| ENSCPOG00000022018_CDS | SYNPO2L | CDS | 5 |
| ENSCPOG00000022307_CDS | MYO9B | CDS | 5 |
| ENSCPOG00000022442_CDS | NA | CDS | 5 |
| ENSCPOG00000022470_CDS | LONP1 | CDS | 5 |
| ENSCPOG00000022499_CDS | LRFN2 | CDS | 5 |
| ENSCPOG00000022563_CDS | NFIX | CDS | 5 |
| ENSCPOG00000022698_CDS | KCNC3 | CDS | 5 |
| ENSCPOG00000022750_CDS | Kcnh2 | CDS | 5 |
| ENSCPOG00000023256_CDS | NA | CDS | 5 |
| ENSCPOG00000023380_CDS | KRT22 | CDS | 5 |
| ENSCPOG00000023551_CDS | ATF6B | CDS | 5 |
| ENSCPOG00000023599_CDS | HGFAC | CDS | 5 |
| ENSCPOG00000023751_CDS | CCDC105 | CDS | 5 |
| ENSCPOG00000023828_CDS | CD248 | CDS | 5 |
| ENSCPOG00000024030_CDS | ATCAY | CDS | 5 |
| ENSCPOG00000024198_CDS | PVALB | CDS | 5 |
| ENSCPOG00000024356_CDS | PPP1R18 | CDS | 5 |
| ENSCPOG00000024360_CDS | COL5A3 | CDS | 5 |
| ENSCPOG00000024560_CDS | CCDC124 | CDS | 5 |
| ENSCPOG00000024620_CDS | CHRNE | CDS | 5 |
| ENSCPOG00000024747_CDS | OR2W6P | CDS | 5 |
| ENSCPOG00000024904_CDS | CRX | CDS | 5 |
| ENSCPOG00000025010_CDS | POU5F1 | CDS | 5 |
| ENSCPOG00000025029_CDS | COMP | CDS | 5 |
| ENSCPOG00000025035_CDS | KCP | CDS | 5 |
| ENSCPOG00000025184_CDS | CACNA1A | CDS | 5 |
| ENSCPOG00000025284_CDS | CCKBR | CDS | 5 |
| ENSCPOG00000025338_CDS | ZBTB47 | CDS | 5 |
| ENSCPOG00000025458_CDS | HDAC5 | CDS | 5 |
| ENSCPOG00000025511_CDS | CACNA1G | CDS | 5 |
| ENSCPOG00000025737_CDS | FBLL1 | CDS | 5 |
| ENSCPOG00000025816_CDS | FAM69B | CDS | 5 |
| ENSCPOG00000025895_CDS | CD5 | CDS | 5 |
| ENSCPOG00000025915_CDS | NCOR2 | CDS | 5 |
| ENSCPOG00000025923_CDS | SPTB | CDS | 5 |
| ENSCPOG00000026115_CDS | RASAL1 | CDS | 5 |
| ENSCPOG00000026167_CDS | NHLRC1 | CDS | 5 |
| ENSCPOG00000026213_CDS | AP1M2 | CDS | 5 |
| ENSCPOG00000026420_CDS | PPAP2C | CDS | 5 |
| ENSCPOG00000026434_CDS | WIPF2 | CDS | 5 |
| ENSCPOG00000026620_CDS | DOCK6 | CDS | 5 |
| ENSCPOG00000026663_CDS | SNAI1 | CDS | 5 |
| ENSCPOG00000026798_CDS | STAC2 | CDS | 5 |
| ENSCPOG00000026862_CDS | DVL3 | CDS | 5 |
| ENSCPOG00000026908_CDS | UNC13A | CDS | 5 |
| ENSCPOG00000027068_CDS | FOXA2 | CDS | 5 |
| ENSCPOG00000027074_CDS | KCNH4 | CDS | 5 |
| ENSCPOG00000027117_CDS | PSMG1 | CDS | 5 |
| ENSCPOG00000027253_CDS | ZNF414 | CDS | 5 |
| ENSCPOG00000027434_CDS | KLF4 | CDS | 5 |
| ENSCPOG00000027503_CDS | GPR179 | CDS | 5 |
| ENSCPOG00000023380_tss | KRT22 | TSS | 5 |
| ENSCPOG00000000779_promoter | EGF | promoter | 5 |
| ENSCPOG00000001181_promoter | PDE8B | promoter | 5 |
| ENSCPOG00000001737_promoter | TRIP6 | promoter | 5 |
| ENSCPOG00000001805_promoter | DMRTC2 | promoter | 5 |
| ENSCPOG00000001821_promoter | JSRP1 | promoter | 5 |
| ENSCPOG00000001921_promoter | TOPAZ1 | promoter | 5 |
| ENSCPOG00000002126_promoter | GPBAR1 | promoter | 5 |
| ENSCPOG00000002141_promoter | ISG20 | promoter | 5 |
| ENSCPOG00000002223_promoter | MYH14 | promoter | 5 |
| ENSCPOG00000002437_promoter | ZNF513 | promoter | 5 |
| ENSCPOG00000002758_promoter | COMTD1 | promoter | 5 |
| ENSCPOG00000003295_promoter | JUP | promoter | 5 |
| ENSCPOG00000003360_promoter | ESRP2 | promoter | 5 |
| ENSCPOG00000003881_promoter | ZDHHC3 | promoter | 5 |
| ENSCPOG00000004153_promoter | OAS3 | promoter | 5 |
| ENSCPOG00000004165_promoter | GABRA5 | promoter | 5 |
| ENSCPOG00000004916_promoter | P2rx7 | promoter | 5 |
| ENSCPOG00000005028_promoter | TPRG1L | promoter | 5 |
| ENSCPOG00000005719_promoter | XRCC1 | promoter | 5 |
| ENSCPOG00000005908_promoter | DPEP3 | promoter | 5 |
| ENSCPOG00000005977_promoter | KDM4B | promoter | 5 |
| ENSCPOG00000007251_promoter | OASL | promoter | 5 |
| ENSCPOG00000007590_promoter | TMIE | promoter | 5 |
| ENSCPOG00000008753_promoter | ENPP5 | promoter | 5 |
| ENSCPOG00000009051_promoter | PPP1R1B | promoter | 5 |
| ENSCPOG00000009074_promoter | MFSD12 | promoter | 5 |
| ENSCPOG00000009579_promoter | CCDC88C | promoter | 5 |
| ENSCPOG00000009855_promoter | CNN1 | promoter | 5 |
| ENSCPOG00000010089_promoter | RFX1 | promoter | 5 |
| ENSCPOG00000010146_promoter | MATN1 | promoter | 5 |
| ENSCPOG00000010822_promoter | DOCK2 | promoter | 5 |
| ENSCPOG00000011503_promoter | TFPT | promoter | 5 |
| ENSCPOG00000012077_promoter | KCTD18 | promoter | 5 |
| ENSCPOG00000012677_promoter | H3F3A | promoter | 5 |
| ENSCPOG00000012842_promoter | NDUFA4L2 | promoter | 5 |
| ENSCPOG00000013001_promoter | PLCB2 | promoter | 5 |
| ENSCPOG00000013080_promoter | NGEF | promoter | 5 |
| ENSCPOG00000014290_promoter | MTA2 | promoter | 5 |
| ENSCPOG00000014611_promoter | C22orf15 | promoter | 5 |
| ENSCPOG00000014724_promoter | FHL2 | promoter | 5 |
| ENSCPOG00000014782_promoter | SLC44A4 | promoter | 5 |
| ENSCPOG00000014982_promoter | KIRREL3 | promoter | 5 |
| ENSCPOG00000019570_promoter | FZR1 | promoter | 5 |
| ENSCPOG00000019761_promoter | C19orf66 | promoter | 5 |
| ENSCPOG00000020131_promoter | PLA2G2C | promoter | 5 |
| ENSCPOG00000020227_promoter | ROM1 | promoter | 5 |
| ENSCPOG00000020483_CDS | CUX2 | CDS | 5 |
| ENSCPOG00000020530_promoter | MOB3A | promoter | 5 |
| ENSCPOG00000020690_promoter | NOP2 | promoter | 5 |
| ENSCPOG00000020725_promoter | COL18A1 | promoter | 5 |
| ENSCPOG00000020908_promoter | NA | promoter | 5 |
| ENSCPOG00000021046_promoter | RBP3 | promoter | 5 |
| ENSCPOG00000021273_promoter | CALCOCO2 | promoter | 5 |
| ENSCPOG00000021290_promoter | NA | promoter | 5 |
| ENSCPOG00000021486_promoter | ZC3H18 | promoter | 5 |
| ENSCPOG00000021771_promoter | NA | promoter | 5 |
| ENSCPOG00000022266_promoter | PKDREJ | promoter | 5 |
| ENSCPOG00000022442_promoter | NA | promoter | 5 |
| ENSCPOG00000022766_promoter | NA | promoter | 5 |
| ENSCPOG00000022975_promoter | NA | promoter | 5 |
| ENSCPOG00000023035_promoter | GSE1 | promoter | 5 |
| ENSCPOG00000023144_promoter | MS4A10 | promoter | 5 |
| ENSCPOG00000023182_promoter | NA | promoter | 5 |
| ENSCPOG00000023380_promoter | KRT22 | promoter | 5 |
| ENSCPOG00000023463_promoter | SNCG | promoter | 5 |
| ENSCPOG00000023502_promoter | CCER1 | promoter | 5 |
| ENSCPOG00000023628_promoter | BGN | promoter | 5 |
| ENSCPOG00000023722_promoter | IER2 | promoter | 5 |
| ENSCPOG00000023754_promoter | NA | promoter | 5 |
| ENSCPOG00000023842_promoter | YJEFN3 | promoter | 5 |
| ENSCPOG00000023899_promoter | RSPH6A | promoter | 5 |
| ENSCPOG00000023943_promoter | NA | promoter | 5 |
| ENSCPOG00000024091_promoter | ASS1 | promoter | 5 |
| ENSCPOG00000024113_promoter | C19orf52 | promoter | 5 |
| ENSCPOG00000024344_promoter | NXNL2 | promoter | 5 |
| ENSCPOG00000024345_promoter | SAPCD1 | promoter | 5 |
| ENSCPOG00000024399_promoter | NTNG2 | promoter | 5 |
| ENSCPOG00000024501_promoter | AK4 | promoter | 5 |
| ENSCPOG00000024530_promoter | NA | promoter | 5 |
| ENSCPOG00000024759_promoter | CACNG6 | promoter | 5 |
| ENSCPOG00000025080_promoter | CHD5 | promoter | 5 |
| ENSCPOG00000025334_promoter | ICMT | promoter | 5 |
| ENSCPOG00000025413_promoter | NA | promoter | 5 |
| ENSCPOG00000025428_promoter | MAP2K2 | promoter | 5 |
| ENSCPOG00000025436_promoter | CYP46A1 | promoter | 5 |
| ENSCPOG00000025539_promoter | NA | promoter | 5 |
| ENSCPOG00000025660_promoter | MISP3 | promoter | 5 |
| ENSCPOG00000025665_promoter | KRTAP17-1 | promoter | 5 |
| ENSCPOG00000025911_promoter | ALPI | promoter | 5 |
| ENSCPOG00000026091_promoter | CTSZ | promoter | 5 |
| ENSCPOG00000026203_promoter | NRTN | promoter | 5 |
| ENSCPOG00000026310_promoter | NA | promoter | 5 |
| ENSCPOG00000026363_promoter | TLE6 | promoter | 5 |
| ENSCPOG00000026509_promoter | NA | promoter | 5 |
| ENSCPOG00000026540_promoter | RNMTL1 | promoter | 5 |
| ENSCPOG00000026620_promoter | DOCK6 | promoter | 5 |
| ENSCPOG00000026807_promoter | TRHR | promoter | 5 |
| ENSCPOG00000027015_promoter | TREML2 | promoter | 5 |
| ENSCPOG00000027227_promoter | HSPBP1 | promoter | 5 |
| ENSCPOG00000027361_promoter | CCDC86 | promoter | 5 |
| ENSCPOG00000027500_promoter | NKX63 | promoter | 5 |
| ENSCPOG00000027520_promoter | PDLIM4 | promoter | 5 |

CDS, coding sequence; tss, transcription start side
